# Temporal and geographic analysis of trichinellosis incidence in Chile with risk assessment

**DOI:** 10.1186/s13071-021-04783-6

**Published:** 2021-05-26

**Authors:** Carlos Landaeta-Aqueveque, Salvador Ayala, Denis Poblete-Toledo, Mauricio Canals

**Affiliations:** 1 Facultad de Ciencias Veterinarias, Universidad de Concepción, Vicente Méndez 595, Chillán, Chile; 2 Instituto de Salud Pública de Chile, Santiago, Chile; 3 Programa de Salud Ambiental, Escuela de Salud Pública and Departamento de Medicina, Facultad de Medicina, Universidad de Chile, Independencia 939, 8380453 Santiago, Chile

**Keywords:** Chile, Disease outbreaks, Foodborne diseases, Incidence, Risk assessment, *Trichinella*

## Abstract

Trichinellosis is a foodborne disease caused by several *Trichinella* species around the world. In Chile, the domestic cycle was fairly well-studied in previous decades, but has been neglected in recent years. The aims of this study were to analyze, geographically, the incidence of trichinellosis in Chile to assess the relative risk and to analyze the incidence rate fluctuation in the last decades. Using temporal data spanning 1964–2019, as well as geographical data from 2010 to 2019, the time series of cases was analyzed with ARIMA models to explore trends and periodicity. The Dickey-Fuller test was used to study trends, and the Portmanteau test was used to study white noise in the model residuals. The Besag-York-Mollie (BYM) model was used to create Bayesian maps of the level of risk relative to that expected by the overall population. The association of the relative risk with the number of farmed swine was assessed with Spearman’s correlation. The number of annual cases varied between 5 and 220 (mean: 65.13); the annual rate of reported cases varied between 0.03 and 1.9 cases per 10^5^ inhabitants (mean: 0.53). The cases of trichinellosis in Chile showed a downward trend that has become more evident since the 1980s. No periodicities were detected via the autocorrelation function. Communes (the smallest geographical administrative subdivision) with high incidence rates and high relative risk were mostly observed in the Araucanía region. The relative risk of the commune was significantly associated with the number of farmed pigs and boar (*Sus scrofa* Linnaeus, 1758). The results allowed us to state that trichinellosis is not a (re)emerging disease in Chile, but the severe economic poverty rate of the Mapuche Indigenous peoples and the high number of backyard and free-ranging pigs seem to be associated with the high risk of trichinellosis in the Araucanía region.

**Supplementary Information:**

The online version contains supplementary material available at 10.1186/s13071-021-04783-6.

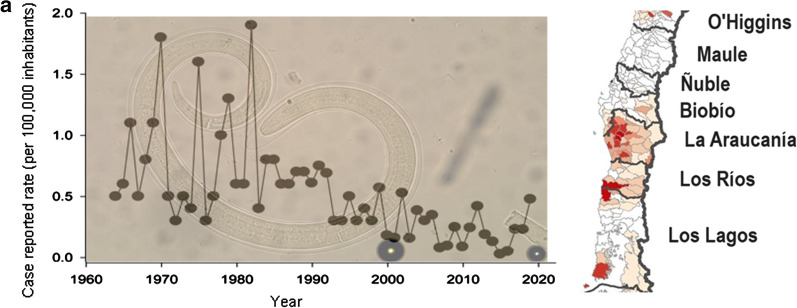

Trichinellosis is a foodborne disease with a worldwide distribution, and is caused by the *Trichinella* species [1]. Several *Trichinella* species circulating among carnivore and omnivore vertebrates have been described. These nematodes are transmitted by meat consumption to humans [1]. Trichinellosis is mainly associated with the household slaughter of pigs (*Sus scrofa domestica* Linnaeus, 1758) or the consumption of game animals without veterinary inspection, especially when the meat has been poorly cooked [2–4]. After consumption, the infection can vary from asymptomatic to lethal, and may present with systemic symptoms associated with the circulation of larvae and infection of the muscles [3, 5].

Currently, there are 13 genotypes of *Trichinella* around the world, 10 of which are recognized as different species [1, 6]. In South America, *Trichinella* has been detected in Brazil, Ecuador (via antibody detection), Bolivia, Argentina, and Chile (via larvae isolation), and most studies have focused on the domestic cycle; Argentina and Chile were found to have the largest number of human cases [7–9]. Two *Trichinella* species have been reported circulating in this continent, mainly in Argentina: *T. spiralis* Owen, 1835 and *T. patagoniensis* Krivokapich et al. 2012 [7]. Conversely, *T.* *spiralis* is the sole species to be reported in Chile, and while studies on wild animals have increased recently [10–15], the domestic cycle was fairly well-studied in previous decades [8], but has been neglected in recent years. Thus, the aim of this study was to analyze geographically the incidence of trichinellosis in Chile. In particular, the goal was to assess the relative risk, as well as to analyze the incidence fluctuations of this disease over the past few decades, assessing the presence or absence of cyclic tendencies.

Trichinellosis is a disease that requires mandatory and immediate reporting to public health authorities of Chile. Those reports pass through several institutions, from local to national levels of the Ministry of Health. In addition, some regulatory laws support the transparency of this information, making it possible to request non-sensitive information regarding this disease.

To analyze the time series of trichinellosis, the information of yearly cases from 1964 to 2019 was obtained from the yearly reports of mandatory notifiable diseases. For spatial analyses, cases spanning 2010–2019 were obtained from the Regional Secretaries of the Ministry of Health for each commune (the smallest geographical administrative sub-division), as per the requirements for this transparent system. When information was not completed through the transparent system, reports from the Instituto de Salud Pública de Chile were used to provide more comprehensive information, but it only featured details from the administrative regions (see Additional file 1: Table S1 in Supporting Information for case details per commune and year).

The yearly time series of cases spanning 1964–2019 was analyzed with autoregressive integrated moving average (ARIMA) models, which examined overall trends and periodicity. The autocorrelation and partial autocorrelation functions were used to select the model. The Dickey-Fuller test was used to study trends, and the Portmanteau test was used to study white noise from the residuals in the model. The 2010–2019 series was used to study the absolute and relative risk by locality. The Besag-York-Mollie (BYM) model was used to make Bayesian maps of relative risks to those expected by population size [16], using WinBUGS and ArcGIS software. The BYM model assumes that the number of cases (*O*_*it*_) in area *i* and period *t* follows a Poisson distribution with the mean:

$$m_{it} = e_{it} \cdot r_{it} ,$$

where *r*_*it*_ is the relative risk and *e*_*it*_ is the expected number of cases, with *e*_*it*_ depending on non-spatial random variation (*U*_*it*_) and the spatially structured variability (*S*_*it*_: neighborhood structure; [16–18]. The relative risk is given as:

$$r_{it} = ae_{it} + U_{it} + S_{it} ,$$

with *a* representing the country-wide rate. The expected number of cases was estimated as:

$$e_{it} = P_{it} I_{t} ,$$

where *P*_*it*_ is the total population of the locality and *I*_*t*_ is the average reported number of cases per 10^5^. The population sizes for each locality and over time were obtained from the National Institute of Statistics of Chile (INE) [19], and the annually reported cases per 10^5^ inhabitants were obtained from the Database of Notifiable Diseases.

Finally, the association between the relative risk with the number of farmed swine was assessed with Spearman’s correlation. The number of farmed pigs and boars was obtained from 2007 agricultural census data [20], given that no recent data have been published (see Additional file 2: Table S2 in Supporting Information for the detailed number of swine per commune).

From 1964 to 2019, the number of annual cases varied from 5 to 220, with a mean of 65.13 and a standard deviation (SD) of 41.06 cases. The annual rate of reported cases varied between 0.03 (in 2015) and 1.9 cases/10^5^ inhabitants (in 1982), with an average and SD of 0.53 ± 0.41 cases/10^5^ inhabitants (see Additional file 3: Table S3 in Supporting Information for the incidence per year). The annual rate series of reported cases of trichinellosis in Chile shows a downward trend that has become more evident since the 1980s (*R* = −0.59, *F*_1,54_ = 29.5; *P* < 0.001; Fig. 1). This trend was removed by first-order differentiation, resulting in a detrended time series (Dickey-Fuller test = −11.9; *P* < 0.001). An ARIMA model was fitted, obtaining an ARIMA (0, 1, 1) model (see Additional file 4: Table S4 in Supporting Information for the model details). An autocorrelation analysis of errors showed adequate adjustment with a Portmanteau *Q* test = 22.83 (*P* = 0.59). The model then corresponded to a first-order moving average model, indicating a weak dependence on random fluctuations from the previous years.

**Fig. 1:**
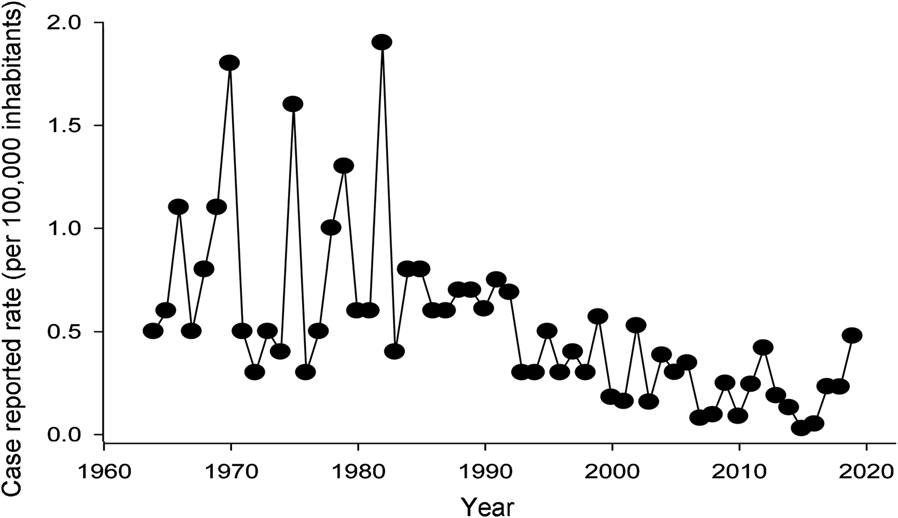
Incidence of trichinellosis in Chile from 1964 to 2019

Communes with at least one case, an incidence rate > 1 cases/10^5^ inhabitants, and a high relative risk (> 1 case/10^5^ inhabitants) were mostly observed in the Araucanía region, followed by the Los Ríos and Los Lagos regions (Fig. 2a, b). The relative risk of the commune was significantly associated with the number of farmed pigs and boar (Spearman’s rho = 0.45; *P* < 0.001).

**Fig. 2:**
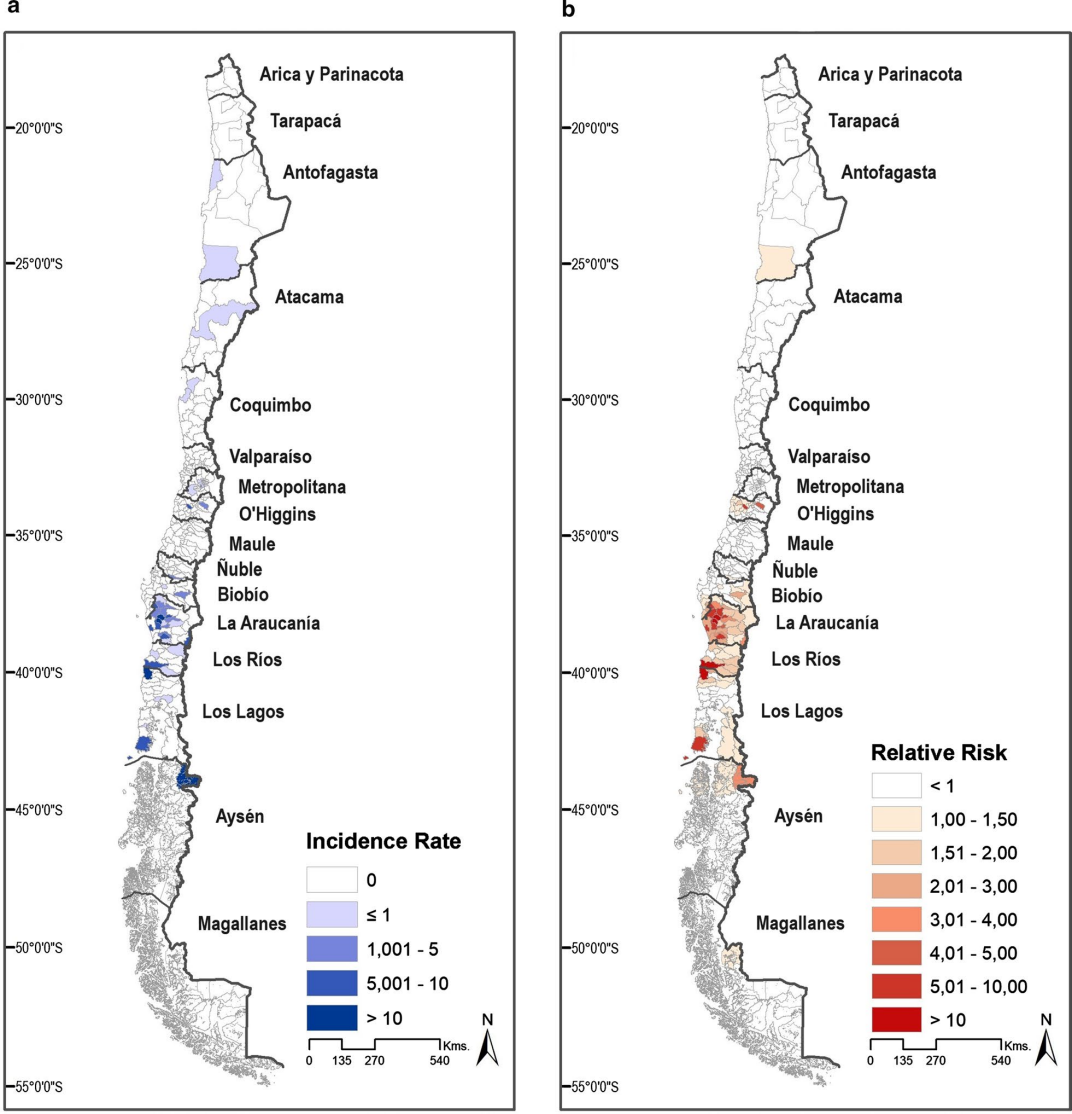
Geographic variation of trichinellosis in Chile. **a** Shows the average incidence of trichinellosis per 100,000 inhabitants between 2010 and 2019. **b** Shows the estimated relative risk of trichinellosis between 2010 and 2019

The decrease in the incidence over time coincides with what has been found in Europe, where the frequency of trichinellosis outbreaks caused by pig consumption had decreased [21]. It was also aligned with the current low global burden of trichinellosis [22], supporting the idea that this is not currently an emerging disease in Chile. During the period spanning 2010–2013, most cases around the world were caused by the consumption of game meat [21]. However, in Chile, most cases are consistently due to domestic pig meat consumption, which corresponds to the association between the level of risk and the number of farmed pigs in a commune; the one exception is that there was a single case of trichinellosis due to wild boar meat consumption [23]. These findings are expected because the hunting of native carnivore mammals is prohibited by law [24], as only the hunting of introduced species is permitted. Alien feral carnivore mammals, such as American minks or feral dogs, are not usually hunted other than for sanitary or ecological control. Thus, wild boar is the sole feral species hunted for consumption in Chile that can act as a direct source of human infection.

The temporal decrease in the incidence rate is in agreement with the reduction in the number of pig farms over the last decade, but not with the reduction of pig production [25], which means that production has been concentrated in larger industrialized farms that face a low risk of *Trichinella* sp. infection [2]. No studies have examined the change in practices among household breeders over time; hence, there is no evidence to support that the decrease in the incidence is due to a change in household-breeding practices. Only a few studies have assessed the knowledge and practices related to *Trichinella* sp. infection in Chile; it was noted that the general population possesses higher knowledge levels and better practices when compared with those for other zoonotic diseases [26, 27]. However, those studies have been performed in communes of the Ñuble region, which have a low relative risk. Since recommendations include focusing on building awareness, among other measures, to prevent trichinellosis outbreaks [28, 29], more studies are needed in other communes with higher risks and incidence rates, particularly those belonging to the Araucanía and Los Ríos regions.

There were no ≤ 3-year cycles observed; thus, no evidence was found to support the presence of regular temporal fluctuations. Rather, a small correlation between long-term decreases in relation to time was found. Few of the reports analyzed in this study included the number of outbreaks and individual cases, as well as the number of cases per commune in a year. In those reports, most cases were due to a few outbreaks (i.e., a sole source of infection), and few cases were individual cases. This suggests that the variations between years could have been due to a few outbreaks that occurred in years with higher incidences, favoring the randomness in temporal variations.

The geographic visualization of the risks does not suggest a latitudinal variation—i.e., the risks did not increase in the south; rather, the highest risks were seen in communes of the Araucanía region, which is explained by the number of pigs bred there. The Araucanía region has the third-largest number of domestic pigs, after the Metropolitan and O’Higgins regions [20]; however, pigs from these latter two regions belong mostly to industrial farms [25]. Therefore, Araucanía is the region with the largest number of household-bred pigs, and it is also the region with the highest proportion of communes with a high relative risk (> 1), which supports the fact that the number of swine farmed in backyards or free-ranging is a significant factor for the risk of trichinellosis. Araucanía is also a region with the largest concentration of Mapuche Indigenous peoples in Chile [19] and is characterized by the most severe economic poverty rate [30], a factor that has been associated with the re-emergence of trichinellosis elsewhere [31].

Feral or wild animals reported to be infected by *Trichinella* in Chile correspond to three cougars (*Puma concolor* Linnaeus, 1771), seven minks, and five wild boars [10, 11, 13–15], most of which were found in the two regions with the highest proportion of communes featuring high relative risks: Araucanía and Los Ríos. Thus, the evidence suggests that there is a small association between human cases and the presence of the parasite in feral animals. However, it is also true that the most sampling efforts to examine those animals have been made in these two regions; hence, more rigorous and unbiased studies are needed to further assess this association.

Thus, our results support the notion that trichinellosis is not a (re)emerging disease in Chile. However, some risk factors could be suggested: the severe economic poverty rate of the Mapuche Indigenous peoples in Chile and the high number of backyard and free-ranging pigs, i.e., pigs reared in non-controlled housing conditions, seem to be associated with the high risk of trichinellosis in the Araucanía region.

## Supplementary Information

**Additional file** **1**: **Table S1**. Details of the number of cases and incidence rates of trichinellosis per commune of Chile and year.

**Additional file** **2**: **Table S2**. Details of the estimation of the relative risk of trichinellosis per commune in Chile.

**Additional file** **3**: **Table S3**. Details of cases of trichinellosis in Chile from 1964 to 2019.

**Additional file** **4**: **Table S4**. ARIMA (0,1,1) model: Time series of the trichinellosis case rate in Chile.

## Data Availability

All data generated or analysed during this study are included in this published article and its supplementary information files.

## Abbreviations

ARIMA: Autoregressive integrated moving average
BYM: Besag-York-Mollie test
INE: National Institute of Statistics of Chile
SD: Standard deviation
*R*: Correlation coefficient
