# Supplementary material for: Temporal and geographic analysis of trichinellosis incidence in Chile with risk assessment
Source: Parasit Vectors. 2021 May 26;14:282. doi: 10.1186/s13071-021-04783-6 (PMC8157648; doi:10.1186/s13071-021-04783-6)
Supplement: Supplementary file 4 — Additional file 4: Table S4. ARIMA (0,1,1) model: Time series of the trichinellosis case rate in Chile. [file 13071_2021_4783_MOESM4_ESM.docx]

**Table S4. ARIMA (0,1,1) model: Time series of the trichinellosis case rate in Chile.**

| Parameter | Coefficient | Standard Error | Z | p-value |
| --- | --- | --- | --- | --- |
| MA(1)^a^ | -0.833 | 0.105 | -7.95 | < 0.001 |
| Constant | 0.349 | 0.026 | 13.53 | < 0.001 |
| Wald χ2 | 63.22 |  |  | < 0.001 |

^a^: Moo Ong first-order average coefficient.
